# Changing how we confirm ngt placement: impact on nutrition delivered

**DOI:** 10.1186/2197-425X-3-S1-A288

**Published:** 2015-10-01

**Authors:** E Segaran, A Hartle, R Leonard

**Affiliations:** Adult ICU, St Mary's Hospital, London, London, United Kingdom

## Introduction

Confirming the position of Nasogastric tubes (NGT) in critical care is a controversial area. The National Patient Safety Agency (NPSA) guidance [1] advocates pH testing although use is limited in ICU due to continuous enteral feeding and proton pump inhibitor therapy. Chest X-ray is therefore commonly used with trainee doctors often being responsible for confirming tube position. Following three incidents of undetected NGT misplacement (outside of our ICU), our trust stipulated a change in practice so that only radiologists or ICU consultants were allowed to confirm placement. We were concerned that this could negatively influence tube confirmation times and enteral nutrition (EN) provision. Cumulative calorie deficits contribute to malnutrition [2]. Under-nutrition in critically ill patients is associated with increased mortality, infections and length of stay [3].

## Objectives

To determine if new guidance for NGT confirmation would influence time off EN and calories delivered.

## Methods

Quality improvement project over two different 4 week time frames, before and after the change in NGT confirmation policy in a general/trauma ICU

## Results

Data was collected on 32 patients, 16 at each data point. See table 1 for demographic details. The majority of NGT placements or replacements occurred in trauma patients. The change in NGT confirmation resulted in significantly longer time off EN and a significant decrease in EN provided (Table 2).

**Table 1:** Patient Demographics.

	Data Point 1 Jan 2014	Data Point 2 Nov 2014
No of Patients	16	16
No of NGT confirmations	36	32
No. of confirmations per patient (median, IQR)	2 (1-3)	2 (1-2.8)
Type of patients n (%)		
Trauma	12 (75)	11 (69)
Medical	3 (19)	5 (31)
Surgical	1 (6)	0
Days data collected (median, IQR)	11 (8-17)	11 (6-13)

**Table 2:** Nutritional data.

	Data Point 1 Jan 2014 Median (IQR)	Data Point 2 Nov 2014 Median (IQR)	P
Hours off EN per episode of NGT (re)placement	5.3 (2-9)	10 (6-16)	0.028
Calorie deficit per episode of NGT (re)placment	402 (363-442)	768 (635-867)	0.04
Calorie deficit per patient for duration of EN received over study duration	2423 (1955-3452)	5660 (4299-7338)	0.00024
% of calories delivered vs. prescribed over study duration	84 (77-87)	71 (62-80)	0.018

## Conclusions

All patients acquired a cumulative calorie deficit over the time they were studied (average of 11 days). The change in NGT confirmation practice had a detrimental effect on time off EN and amount of EN provided. The size of increase in calorie deficit between the two time periods is only partly attributable to the change in NGT confirmation. Other factors influenced EN delivery in our patients, between the two data points such as non-compliance with our fasting guidelines for surgery and airway procedures. We speculate that the change in NGT confirmation may have led to an excessively risk-averse approach in which the problems of cumulative calorie deficit were under-appreciated. The current NGT confirmation practice for ICUs in our organisation is being reviewed. It is proposed once again to permit ICU trainees who have successfully completed relevant competency training to confirm position of NGT on x-ray. We intend to repeat data collection once this change has been implemented.

## Grant Acknowledgement

No external funding was sought for this project.
